# LTP induction within a narrow critical period of immature stages enhances the survival of newly generated neurons in the adult rat dentate gyrus

**DOI:** 10.1186/1756-6606-3-13

**Published:** 2010-04-28

**Authors:** Takashi Kitamura, Yoshito Saitoh, Akiko Murayama, Hiroyuki Sugiyama, Kaoru Inokuchi

**Affiliations:** 1 Mitsubishi Kagaku Institute of Life Sciences, MITILS, 11 Minamiooya, Machida, Tokyo, 194-8511, Japan; 2 Department of Biochemistry, Faculty of Medicine, Graduate School of Medicine & Pharmaceutical Sciences, University of Toyama, 2630 Sugitani, Toyama 930-0194, Japan; 3 Japan Science and Technology Agency, CREST, Kawaguchi 332-0012, Japan; 4 Department of Biology, Graduate School of Science, Kyushu University, Fukuoka 812-8581, Japan

## Abstract

Neurogenesis occurs in the adult hippocampus of various animal species. A substantial fraction of newly generated neurons die before they mature, and the survival rate of new neurons are regulated in an experience-dependent manner. Previous study showed that high-frequency stimulation (HFS) of perforant path fibers to the hippocampal dentate gyrus (DG) induces the long-term potentiation (LTP) in the DG, and enhances the survival of newly generated neurons in the DG. In this study, we addressed whether a time period exists during which the survival of new neurons is maximally sensitive to the HFS. We found that the enhancement of cell survival by HFS was exclusively restricted to the specific narrow period during immature stages of new neurons (7-10 days after birth). Furthermore, the pharmacological blockade of LTP induction suppressed the enhancement of cell survival by the HFS. These results suggest that the LTP induction within a narrow critical period of immature stages enhances the survival of newly generated neurons in rat DG.

## Introduction

Large numbers of new neurons are generated throughout adulthood in the hippocampal dentate gyrus (DG) of many mammals, including rats, monkeys, and humans [1-3]. New neurons are functionally integrated into pre-existing neuronal circuits [4-7] and contribute to learning and memory [8], particularly the systems consolidation of memory [9]. A subset of newly generated granule cells is selected to survive, and the others are eliminated through programmed cell death within a month [10-14]. The survival rate of new neurons is regulated in an experience-dependent manner, such as environmental enrichment, running wheel exercises, and learning contributing to survival [15-18]. Previous study shows that high-frequency stimulation (HFS) of perforant path fibers to the DG induces long-term potentiation (LTP) in the DG and enhances the survival of newly-born neurons in the DG [19]. In this study, we addressed whether a time period exists during which the survival of new neurons is maximally sensitive to HFS. Furthermore, to understand the causal relationship between LTP induction and the survival, we used an antagonist of the N-methyl-D-aspartate (NMDA)-type glutamate receptor to inhibit the induction of LTP, and under these conditions we examined the effect of HFS on the survival rate of new neurons in the DG of unanesthetized freely moving rats.

## Methods

### Animals

We examined male Wistar ST rats approximately 20-25 weeks of age. All procedures involving the use of animals were conducted in compliance with the guidelines of the National Institutes of Health and were approved by the Animal Care and Use Committee of the Mitsubishi Kagaku Institute of Life Sciences.

### Immunohistochemistry

**Figure 1:**
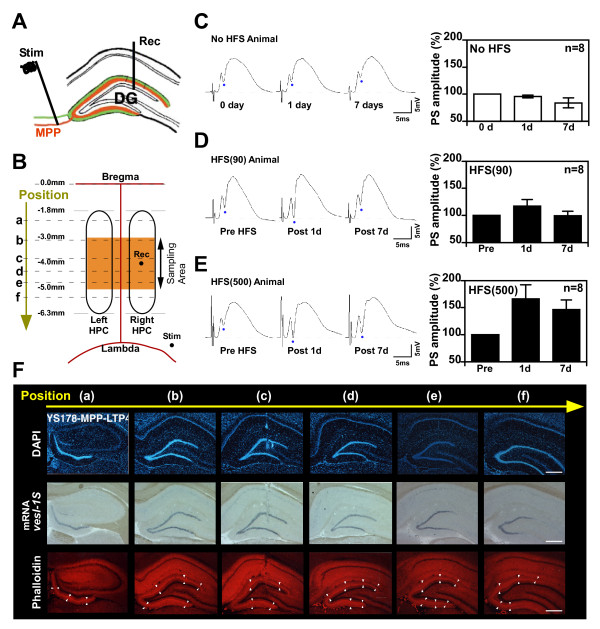
**Sampling region for evaluating the effect of HFS on neurogenesis in the DG**. **(A) **Anatomical organization of the entorhinal-hippocampal dentate gyrus (DG) pathway. Abbreviations: Rec, recording electrode; Stim, stimulating electrode; MPP, medial perforant path fibers (orange); LPP, lateral perforant path fibers (green). **(B) **Overhead schematic view of a rat brain. The orange area indicates the sampling area (-3.0 mm to -5.0 mm relative to bregma) for the assay to evaluate neurogenesis. HPC, Hippocampus. **(C) **PS amplitudes of the DG at 0 day and 1 day and 7 days from start fEPSP recording (no HFS, test pulse only). **(D, E) **PS amplitudes of the DG at pre-HFS, and 1 day and 7 days after the HFS treatment in HFS (90) (D) and HFS (500) (E) groups. Typical fEPSP traces evoked before HFS (left) and 1 day after HFS (middle) and 7 days after HFS (right) are also shown. Error bars indicate SEM. The number of animals used is indicated by "n". **(F) **Nuclear staining by DAPI (upper photos), *vesl-1S *mRNA by in situ hybridization (middle photos), and F-actin staining by phalloidin (lower photos) through the anterior-posterior extent of hippocampus of HFS (500)-treated rat. (a-f) indicate the positions in the hippocampus from the anterior to posterior, and correspond to (a-f) in Fig 1B. Scale bars, 500 μm.

Animals were anesthetized with an overdose of pentobarbital solution and the fully anesthetized animals were perfused transcardially with 0.9% saline, followed by 4% paraformaldehyde (PFA) in phosphate-buffered saline (PBS). Brains were stored in fixative (4% PFA in PBS) for 3 h at 4°C, and then incubated overnight in 30% sucrose, followed by immersion in dry ice powder. A cryostat was used to collect rat brain sections of 20 μm thickness. For BrdU immunohistochemistry, sections were boiled for 10 min, treated with 2 M HCl for 30 min, rinsed in 0.1 M boric acid (pH 8.5) for 10 min, as previously [20]. Sections were blocked with 0.1% BSA or 3% goat serum in PBS containing 0.1% Tween-20 (PBS-T) at room temperature for 1 h. After blocking, tissues were incubated with blocking solution containing rat monoclonal anti-BrdU (1:250; Serotech) and mouse monoclonal anti-NeuN (1:200; Chemicon) antibodies. Sections were then incubated with anti-rat IgG-Cy3 and anti-mouse IgG-AlexaFluor 488 or counterstained with SYTOX GREEN (Invitrogen). Procedures to quantify hippocampal cell proliferation and neurogenesis have been described previously [9]. Briefly, BrdU-labeled cells were sampled in the dorsal DG (-3.0 mm to -5.0 mm relative to bregma, Fig. 1B). Every other 20-μm coronal section was used for counting the number of BrdU-labeled cells (total of 50 sections per rat), using a 40× objective (BX41, OLYMPUS), in a treatment-blinded manner. All BrdU-labeled cells, regardless of size or shape, were counted. A cell was counted if it adjoined SGZ or was placed in SGZ or GCL (exclude the hilus region).

### Dentate gyrus LTP in unanesthetized freely moving rats

We used a surgical procedure described previously [9,21-23]. Briefly, a bipolar stimulating electrode and a monopolar recording electrode made of tungsten wire were positioned stereotaxically to selectively stimulate the medial perforant pathways (MPP) and projections. The electrode stimulating the MPP fibers was positioned 8.7 mm posterior, 5.3 mm lateral, and 5.3 mm inferior to bregma. A recording electrode was implanted ipsilaterally 4.0 mm posterior, 2.5 mm lateral, and 3.8 mm inferior to bregma (Fig. 1A, B). Rats were allowed to recover for at least 2 weeks in individual home cages. Following recovery, input/output curves reflecting evoked field excitatory postsynaptic potentials (fEPSP) as a function of current intensity (0.1-1.0 mA) were collected for each rat. We then selected a current intensity that evoked 60% of the maximum population spike amplitude for all stimulations, and kept the intensity constant throughout the experiment. There was no difference in current intensity of test pulse stimulation between all experimental groups (data not shown). Test stimuli were delivered at 20-s intervals to record the fEPSP. We used strong and weak experimental stimuli. HFS (500), the strong tetanic stimulus (biphasic square wave form, 200 μs pulse width), consisted of 10 trains with 1-min intertrain intervals. Each train consisted of 5 bursts of 10 pulses at 400 Hz, delivered at 1-s interburst intervals. The weak tetanic stimulation, HFS (90), consisted of 6 trains at 10-s intertrain intervals. Each train consisted of 15 pulses at 200 Hz (biphasic square wave form, 200 μs pulse width).

### Statistical analyses

All data are presented as mean ± SEM. The number of animals used is indicated by "n". Comparisons between two-group data were analysed by unpaired Student's t-test or paired t-test. Multiple group comparisons were assessed using a one-way analysis of variance (ANOVA), followed by the post-hoc Scheffe's test when significant main effects were detected. The null hypothesis was rejected at the P < 0.05 level.

## Results

### Sampling region for evaluating the effect of HFS on neurogenesis in the DG

We examined LTP in the dentate gyrus of freely moving unanesthetized rats. We monitored population spike (PS) amplitudes in the DG after the delivery of HFS (Fig. 1C, D, E). When the weak HFS (90) was delivered, the potentiation of PS amplitudes persisted for 1 d but decayed to the basal level within 7 d (Fig. 1D), as described previously [22]. In contrast, the potentiation of PS amplitudes in the DG persisted for at least 7 d when the strong HFS (500) was delivered (Fig. 1E). HFS (500) also induces the persistent increase in slope of fEPSP [9]. To clarify the dentate area that is affected by HFS (the region where LTP may occur), we examined the expression of *vesl-1S/Homer-1a *mRNA. *vesl-1S/Homer-1a *is a neural activity-regulated gene [21,24,25] whose expression is positively regulated by LTP induction. We also stained sections for phalloidin, a specific probe for F-actin, as an index of LTP; LTP is accompanied by enhanced F-actin content in spines that is essential for LTP maintenance [22]. We sampled the brain 45 min after applying HFS (500). The brain was cut by coronal section through the anterior-posterior extent of DG. We detected the expression of *vesl-1S *mRNA in nuclei of granule cells and the enhancement of phalloidin reactivity in the middle molecular layer (MML) in the dorsal DG area and partially in the ventral DG area (Fig. 1F). In control rats without HFS treatment, there are no detections in the expression of *vesl-1S *mRNA in nuclei of granule cells [21] and in the enhancement of phalloidin reactivity in the MML [22]. We therefore chose the dorsal DG area (from -3.0 mm to -5.0 mm relative to bregma) as the sampling region for evaluating the effect of HFS on neurogenesis in the DG (Fig. 1B).

### HFS enhances cell proliferation in the DG

**Figure 2:**
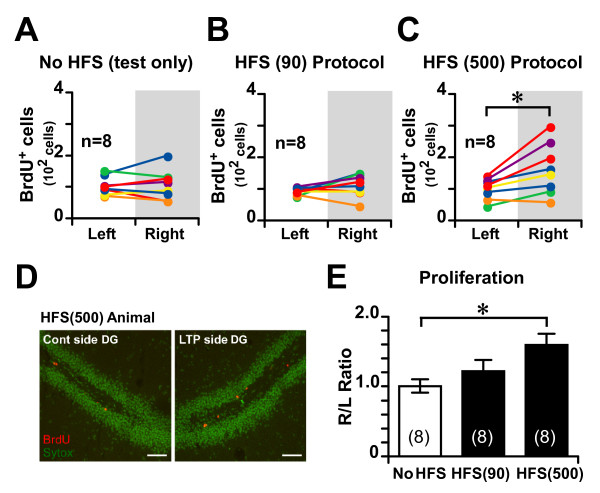
**HFS enhances cell proliferation in the DG**. **(A, B, C) **The number of BrdU-labeled cells in the sampling area of the DG in the HFS side (right) and the contralateral side (left). **(D) **BrdU (red) immunoreactivity 1 day after a BrdU injection into the DG of an HFS (500)-treated rat. Nuclei (green) are stained with SYTOX Green. Scale bars, 100 μm. **(E) **Right/left (R/L) ratio of the number of BrdU-labeled cells. * *P *< 0.05, error bars indicate SEM. The number of animals used is indicated by "n" (A, B, C) or is given in parentheses (E).

Previous studies have shown that HFS increases cell proliferation in the rat DG [19,26,27]. First we examined the effect of HFS on cell proliferation in our experimental conditions. We induced LTP by HFS (500) or HFS (90), and 4 d later injected bromodeoxyuridine (BrdU; 150 mg/kg, i.p., Sigma). Twenty-four hours after the BrdU injection, the rats were perfused. Consistent with previous studies [19,27], HFS (500) significantly increased the number of BrdU-labeled cells on the HFS-treated side (right), when compared with the contralateral (left) side (paired t-test: *t*_7 _= -3.29, *P *< 0.02) (Fig. 2C, D). There was no difference between the sides in the test-pulse group (no HFS) (paired t-test: *t*_7 _= -0.23, *P *> 0.8) (Fig. 2A). Interestingly, HFS (90), which induces transient LTP (Fig. 1D), did not enhance the number of BrdU-labeled cells when compared with the contralateral side (paired t-test; *t*_7 _= -1.46, *P *> 0.1) (Fig. 2B), suggesting that strong HFS enhances cell proliferation in the DG, but weak HFS does not. To directly compare the effect of HFS on cell proliferation in the three groups, we calculated the ratio of BrdU-labeled cells in the ipsilateral side to the number in the contralateral side (the R/L ratio) within each animal. The R/L ratio differed significantly between the test-pulse and HFS (500) groups (*P *< 0.02, ANOVA, post-hoc Scheffe's test) (Fig. 2E). There was no difference between the test-pulse and HFS (90) groups in R/L ratio (*P *> 0.5, ANOVA, post-hoc Scheffe's test) (Fig. 2E). From these results, we selected the HFS (500) protocol to investigate the effect of HFS on cell survival in the DG.

### LTP induction within a narrow critical period enhances cell survival in the DG

**Figure 3:**
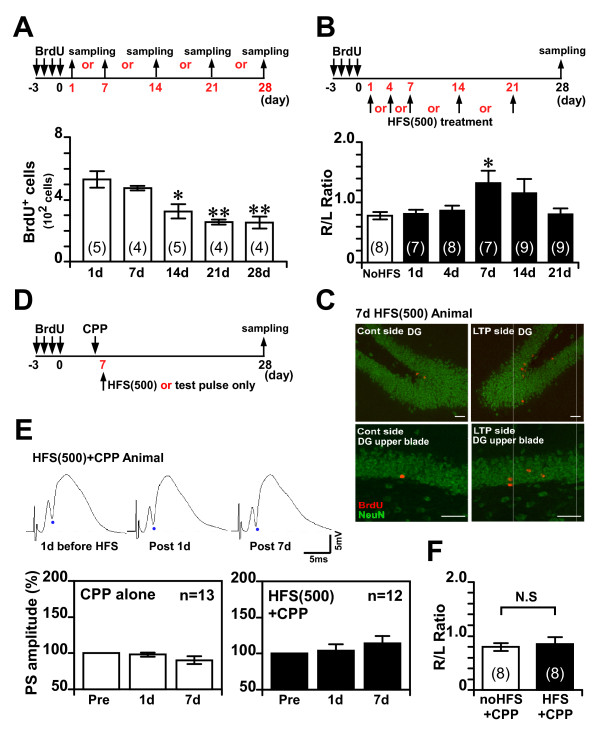
**LTP induction within a narrow critical period enhances cell survival in the DG**. **(A) **Top: Experimental schedule. Bottom: Number of BrdU-labeled cells at various time points after BrdU injection. BrdU was given twice a day for 4 consecutive days, and cells were counted in the sampling areas 1, 7, 14, 21, and 28 days after the last injection of BrdU. **(B) **Top: Experimental schedule for the effect of HFS (500) on cell survival. Bottom: Right/left (R/L) ratio of the number of BrdU-NeuN double-labeled cells at the indicated times after HFS treatment. **(C) **Immunoreactivity for BrdU (red) and NeuN (green) 28 days after the last BrdU injection in the DG of a rat treated with HFS (500) 7 days after the last BrdU injection. Scale bars, 50 μm. **(D) **Experimental schedule for the effect of CPP on the enhancement of survival by HFS. **(E) **Top: Typical fEPSP traces evoked 1 days before HFS (left), 1 days after HFS (middle), and 7 days after HFS (right). Bottom: PS amplitudes of the DG before HFS (Pre) and 1 day and 7 days after HFS (500) in the CPP-alone and CPP-HFS groups. **(F) **R/L ratio of the number of BrdU-NeuN double-labeled cells in the CPP-alone and CPP-HFS groups. * *P *< 0.05, * * *P *< 0.01, N.S indicates 'Not Significant'. Error bars indicate SEM. The number of animals used is indicated by "n" (E) or is given in parentheses (A, B, F).

To study the time course of the survival of newly generated cells in the DG of 20-week-old rats, we determined the numbers of BrdU-labeled cells in the DG at 1, 7, 14, 21, and 28 days after the last injection of BrdU (Fig. 3A). Consistent with previous studies, we found a significant decrease in the number of BrdU-labeled cells within 2 weeks after the last BrdU injection in 20-week-old rats (1 d vs. 14 d, *P *< 0.05; 1 d vs. 21 d, *P *< 0.01; 1 d vs. 28 d, *P *< 0.01, ANOVA, post-hoc Scheffe's test) (Fig. 3A). Next, to address the issue of whether a time period exists during which the survival of new neurons is maximally sensitive to HFS treatment, we injected rats with BrdU twice a day for 4 d; delivered HFS (500) 1, 4, 7, 14, or 21 days later; and then quantified the number of surviving BrdU-labeled cells 28 days after the last BrdU injection (Fig. 3B), because by 4 weeks after their birth, the new cells have differentiated into mature neurons and their numbers have become stable [11]. To detect the surviving BrdU-labeled neurons, we performed immunohistochemistry for BrdU and the neuronal marker NeuN, and quantified the number of BrdU-NeuN double-labeled neurons. Interestingly, only the HFS (500) applied 7 d after the last BrdU injection significantly increased the R/L ratio of the BrdU-NeuN double-labeled cells, when compared with the test-pulse group (*P *< 0.05, ANOVA, post-hoc Scheffe's test) (Fig. 3B, C). Other protocols of HFS (500) delivery did not significantly increase the R/L ratio (*P *> 0.7, ANOVA, post-hoc Scheffe's test), although HFS (500) applied 14 d after the last BrdU injection resulted in a numerically higher R/L ratio. An analysis of cell type revealed that the HFS (500) did not change the ratio of differentiation to neuron (BrdU^+^NeuN^+ ^cells/BrdU^+ ^cells), when compared with the test-pulse group (test-pulse group, 49.4% ± 2.3%; 7d HFS group, 51.1% ± 2.6%, *t*_13 _= -0.50; *P *> 0.6). These results clearly indicate that HFS (500) significantly increases the survival of newly generated neurons but not the ratio of differentiation to neuron, and that there is a narrow critical period (7-10 days after birth, because we injected BrdU for 4 days) for the enhancement of cell survival by HFS (500).

Finally, to address whether the enhancement of cell survival by HFS (500) is due to the induction of LTP within the narrow critical period, we used 3-(2-carboxypiperazin-4-yl) propyl-1-phosphonic acid (CPP), which is a selective NMDA receptor antagonist, to block the induction of LTP in the perforant path-DG synapses (Fig. 3D). The injection of CPP (10 mg/kg, i.p., TOCRIS) 2 hours before HFS (500) clearly blocked the LTP induction in rat DG (Fig. 3E). We examined the effect of the pharmacological blockade of LTP induction on cell survival when HFS (500) was applied 7 d after the last BrdU injection (Fig. 3D). We observed no difference between the group with CPP injection alone and the CPP-HFS group in the R/L ratio of the number of BrdU-NeuN double-labeled cells (t-test; *t*_13 _= -0.40, *P *> 0.6) (Fig. 3F), indicating that injecting CPP before HFS suppressed the ability of HFS (500) to enhance the survival of newly generated neurons. CPP injection alone 7 days after the last BrdU injection did not affect cell survival in DG of control rats (no HFS treatment, measured by the number of BrdU^+^NeuN^+ ^cells 28 days after last BrdU injection, CPP(-) group, 178 ± 13 cells; CPP(+) group, 158 ± 17 cells, *t*_14 _= 0.93; *P *> 0.3).

## Discussion

Consistent with previous reports [19,26,27], we observed that HFS (500) of perforant path fibers to the DG increases the cell proliferation in the DG. Interestingly, HFS (90), which elicited relatively short LTP that persisted for 24 hr but decayed to the basal level within 7 days, did not enhance the proliferation in the DG. Previous report shows that the stimulation protocol (288 pulse, 400 Hz), which is intermediate between the HFS (500) and HFS (90), enhances the cell proliferation in the DG [19]. The protocol is similar to the HFS (300) (300 pulse, 400 Hz), which results in 7 days LTP [22]. Taken together, these results suggest that the protocols of HFS which induces persistent LTP (1 week~) in the DG are required for the enhancement of cell proliferation.

We observed that HFS of perforant path fibers to the DG enhances the survival of newly born neurons in the DG, as previously reported [19]. The effect of HFS was restricted to a specific narrow period (7-10 days after cell birth) during the immature stages of new neurons. Furthermore, this enhancement was blocked by CPP, which completely blocked the induction of LTP in the DG. These results strongly suggest that LTP induction within a narrow critical period during neuronal development enhances the survival of newly generated neurons in the adult rat DG.

How does LTP induction in the DG enhance the survival of newly generated neurons? Taking advantage of *in vivo *LTP paradigm, we selectively applied HFS to the medial perforant path fibers (MPP) and elicited LTP specifically at the synapses in the middle molecular layer (MML) [22]. Under these conditions, we examined here the effect of HFS on the survival of 7-10 days-old cells in the DG. Using the same rat strain with similar ages, ca. 20-weeks old, we previously showed that the apical dendrites of 7-10 days-old cells do not reach MML nor do they have dendritic spines [28]. These observations strongly suggest that 7-10 days-old cells do not make synapses in MML nor receive glutamatergic input from MPP. Given the timing of dendritic growth and spine formation in the MML, we speculate that HFS (500) may activate the NMDA receptors of pre-existing mature neurons, which in turn may release the secreted factors from the mature neurons to extrinsically modulate the survival of young neurons in the 7 to 10 d after birth [12,29,30]. Indeed, HFS (500) increased not only the number of surviving BrdU^+^NeuN^+ ^cells but also the number of surviving BrdU^+^NeuN^- ^cells; these are non-neuronal cells (Fig. 3), suggesting that HFS (500) may trigger the release of general survival factor(s). Activin, a member of the transforming growth factor-β superfamily, induces numerous cell differentiations and promotes cell survival [31]. Previously, we and others found that the HFS treatment increases the level of activin β A mRNA in mature granule neurons of the DG in an NMDA receptor-dependent manner [32,33]. Furthermore, our transgenic approach revealed that the activin signaling is crucial for the survival of newly generated neurons in the adult DG [34,35]. Our experiments with hippocampal culture also showed that activin treatment increases the population of surviving Prox-1-positive cells at DIV21 (Prox-1 is a marker of granule cells of dentate gyrus), whereas follistatin, an inhibitor of activin signalling pathway, decreases the population [36]. Based on these studies, the HFS-induced release of activin β A from mature granule neurons may specifically affect the immature neurons during this narrow period, and then may enhance the survival rate of new neurons in adult DG. Finally, we would like to point out a recent electrophysiological study that suggests that at 10 days after birth, small population of new cells (9.3%) begin to receive the glutamatergic input from the perforant path, [37]. Thus, at present we do not completely exclude the possibility that HFS directly acts on the 7-10 days-old cells for survival. Further investigation is required to elucidate the mechanisms through which HFS regulates the survival of new cells.

Previous studies have shown that some form of hippocampus-dependent learning and the experiences of an enriched environment also affect the survival of new neurons at early immature stages in the DG [16-18]. Thus, there is a check point during the early immature stages of neurons that responds to experience to determine whether new neurons in the adult hippocampus would survive or die experience-dependently in later stages of development.

## Competing interests

The authors declare that they have no competing interests.

## Authors' contributions

This study was conceived and designed by T.K. and K.I. Electrophysiological experiments were conducted by Y.S and T.K. Histological experiments were conducted by T.K and A.M. The original concept of this study was conceived by T.K., Y.S., H.S. and K.I. The manuscript was written by T.K and K.I. The entire project was supervised by K.I. All authors read and approved the final manuscript.
